# An improved method for extraction of soil fungal mycelium

**DOI:** 10.1016/j.mex.2023.102477

**Published:** 2023-11-07

**Authors:** Abdallah Awad, Rodica Pena

**Affiliations:** Forest Botany and Tree Physiology, University of Göttingen, Büsgenweg 1, Göttingen 37077, Germany

**Keywords:** Extraradical mycelium, Density gradient, Hyphal length, Temperate forests, Extraction of soil fungal mycelium

## Abstract

Fungal mycelium is a major component of the soil microbiome. The soil hyphosphere represents a complex and dynamic niche for specific microorganisms, where multitrophic interactions occur, affecting ecosystem processes. However, extracting fungal mycelium from the soil to enable its taxonomical, chemical, and structural characterisation is challenging in the absence of a fast, efficient, and low-cost procedure. In this study, an old method (Bingle and Paul 1985), based on successive soil wet filtrations and density gradient centrifugation, was improved and tested in three different soil types (silty clay, silty clay loam, and loamy sand). The improved method reduced the number of filtrations by about five times and the centrifugation time from 40 min to 1 min. It avoided using any chemical substance which may impair further chemical analyses or DNA isolation and amplification. The method efficiency was about 50 % in the clay and 23 % in the sandy soils. However, a pre-step consisting of removing the fine-root fragments and other debris under the stereomicroscope may increase the method efficiency to more than 65 %, independent of the soil type.

• A simple, efficient, and low-cost method suitable for extracting soil mycelium from a large number of samples.
• The protocol includes successive soil wet filtrations and sucrose gradient centrifugation.
• The method efficiency increases if the fine-root fragments and other debris are previously removed from the soil.

## Graphical abstract

**Figure:**
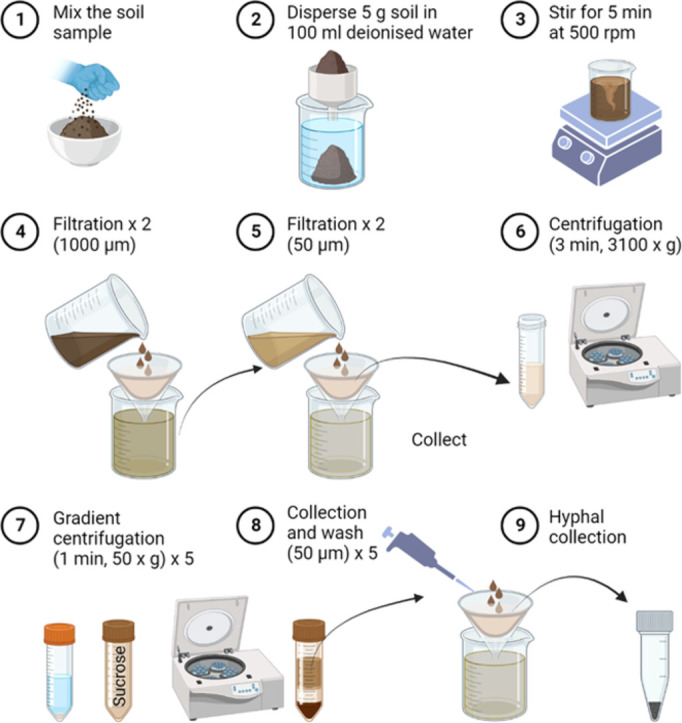

Specifications table

**Table:** 

Subject area:	Agricultural and Biological Sciences
More specific subject area:	*Soil microbiology*
Name of your method:	*Extraction of soil fungal mycelium*
Name and reference of original method:	*Bingle, W.H., Paul, E.A., 1986. A method for separating fungal hyphae from soil. Canadian Journal of Microbiology 32, 62–66. doi:*10.1139/m86-012
Resource availability:	*Reagents and Equipment are listed in the Materials section*

## Method details

 

## Background

Soil filamentous fungi are crucial in carbon (C) and nutrient cycling in terrestrial ecosystems [1]. They decompose organic material to obtain C and other nutrients [2], channel the plant-fixed C to the soil (mycorrhizal fungi), and contribute to soil C storage through their residues and conversion of C-containing biomolecules into decay-resistant supramolecular complexes [3,4]. Soil fungi move C and nitrogen (N) within the forest soil profile [5] and nutrients from the soil to plants [6], improving plant productivity. Fungal mycelia represent 45–60 % of soil microbial biomass [7]. In temperate and boreal forests, they account for more than 3500 kg ha^−1^ with a turnover rate of 25 days to 1 year [8], [9], [10]. The hyphosphere, consisting of the hyphal surface and the hyphae-adjacent soil, which is influenced by hyphal exudates, is a very complex and dynamic zone of multitrophic interactions [11]. It forms a niche for specific microorganisms (e.g., bacteria) that can solubilise organic or inorganic nutrients having a large influence on the soil nutrient economy [12], [13], [14], [15].

In the last decades, an unprecedented amount of fungal taxon-specific molecular sequences from environmental samples has become available through the employment of high-throughput DNA sequencing [16]. However, the fungal sequence assignment to taxon functional identity is minimal [17] as currently less than 5 % of soil fungi have been isolated, cultivated and functionally described [18]. Molecular DNA metabarcoding data, combined with functional analyses, may enable functional characterisation at the level of fungal metacommunities, bringing no or only minimal information on the functions of specific community components [16,19]. A more precise sampling, consisting of soil mycelium rather than bulk soil, may refine DNA metabarcoding, further allowing the estimation of more accurate links between soil fungal biodiversity and ecosystem processes whilst at the same time addressing the hyphosphere complexity.

Sample preparation is recognised as one of the most critical steps in the DNA metabarcoding and sequencing of soil fungal communities [16,[20], [21], [22]]. The soil fungal community can be enriched with specific taxa depending on how the sample is processed. The vast majority of studies which specifically address the soil fungal communities used the DNA extracted from the soil with no previous processing but removing coarse roots and stones [23], [24], [25]. Some authors sieved the soil through a 2 mm-mesh sieve. Therefore, the soil-extracted DNA may belong not only to the fungal mycelium in soil but fungi associated with fine roots, fragments of mycorrhizal root tip mantle, small decaying wood material, or fungal spores. Fungal communities of all these fractions differ from soil mycelium [26,27], inflating the soil fungal metacommunities and biasing any functional estimates [28].

After fungal cell death, remnant DNA is often sorbed to the soil matrix, eluding the microbial decay and contributing to the soil DNA pool that is commonly extracted and amplified to assess the soil fungal communities [29]. Hence, extracting and amplifying the DNA from soil material also results in the overrepresentation of fungal communities due to relic DNA, which can represent more than 40 % of the fungal molecular sequences (internal transcribed spacer, [29]).

As technology and research have progressed, the functional and taxonomical exploration of soil mycelium have been found to be difficult in the absence of reliable methods to isolate the mycelium from the soil.

Bingle and Paul [30] have provided a method to separate the fungal hyphae from soil particles using successive wet filtrations and glycerol gradient density centrifugation. In principle, this method is recommended for collecting soil mycelium for chemical and nutrient analysis [31], but its applicability for nucleic acid isolation is possibly hampered by using sodium pyrophosphate solution in all its steps. Sodium pyrophosphate is required to increase the soil dispersal, but it can be an inhibitor of the Taq polymerase and PCR reaction [32,33]. Moreover, the method is time-consuming since it involves a high number of filtration steps, preventing its use for a large number of samples concomitantly.

In this study, we improved Bingle and Paul's method for soil fungal mycelium extraction by firstly excluding from the procedure any chemical compounds which may impair the subsequent DNA isolation and amplification. Secondly, we reduced the number of filtration steps by five times and, finally, reduced the centrifugation time from 40 min to 1 min.

We tested the reliability of the method by extracting soil mycelium from three soil types, differing in structure, chemistry, and microbial biomass. The efficiency of the method was controlled by microscopic analysis of all discarded fractions and measuring the hyphal lengths of the initial soil sample, extracted mycelium, and discarded soil residues.

### Soil collection and sample preparation

Soil samples were collected in 27 experimental plots (100 × 100 m) located in European beech (*Fagus sylvatica* L.) dominated forests in three regions of Germany: the UNESCO Biosphere-Reserve Swabian Alb (Alb, 7 plots) in the southwest; the region Hainich-Dün (Hai, 10 plots), including the National Park Hainich, in central Germany; and the UNESCO Biosphere-Reserve Schorfheide-Chorin (Sch, 10 plots) in the northeast Germany.

The three regions differ in climatic conditions, with mean annual temperatures ranging from 6 to 8.5 °C (from Alb to Sch) and mean annual precipitation from 500 to 1000 mm (from Sch to Alb). The soil types differ amongst the regions, with clay contents, pH values, and microbial biomass decreasing from Alb to Sch (Table 1). The plots belonged to the infrastructure of the DFG-Biodiversity Exploratories project (https://www.biodiversity-exploratories.com, [34].

**Table 1:** Soil properties of the three study sites (after [34,40]).

Soil properties	Swabian Alb (Alb)	Hainich-Dün (Hai)	Schorfheide Chorin (Sch)
Soil type	Cambisol / Leptosol	Luvisol	Cambisol
pH	5.0 ± 1.0	5.0 ± 1.0	3.0 ± 0.1
Soil bulk density (g cm^−3^)	0.82 ± 0.01	0.91 ± 0.05	1.14 ± 0.05
Clay (g kg^−1^)	496 ± 105	301 ± 99	45 ± 19
Sand (g kg^−1^)	60 ± 46	58 ± 18	871 ± 61
C/N ratio	13 ± 1	13 ± 1	18 ± 3
Microbial biomass (µg *g*^−1^)	737 ± 220	449 ± 214	123 ± 38

In each plot, seven soil cores with a diameter of 0.08 m and depth of 0.10 m were collected at equal distances along two 40 m long transects plotted from north to south and west to east, respectively. Before coring, the surface organic layer was removed, resulting in the collection of the top mineral soil comprising the A horizon. All samples of a plot were mixed and well homogenised, giving one composite sample per plot. The soil was sieved through a 2-mm sieve and inspected to find and remove the remaining root fragments. A 10.0 g aliquot of soil was taken from each sample and divided into two equal parts. One part was used for mycelium extraction and another for measuring the total hyphal length. The samples were stored at −20 °C for further analysis.

### Extraction procedure of soil mycelium

**Fig. 1:**
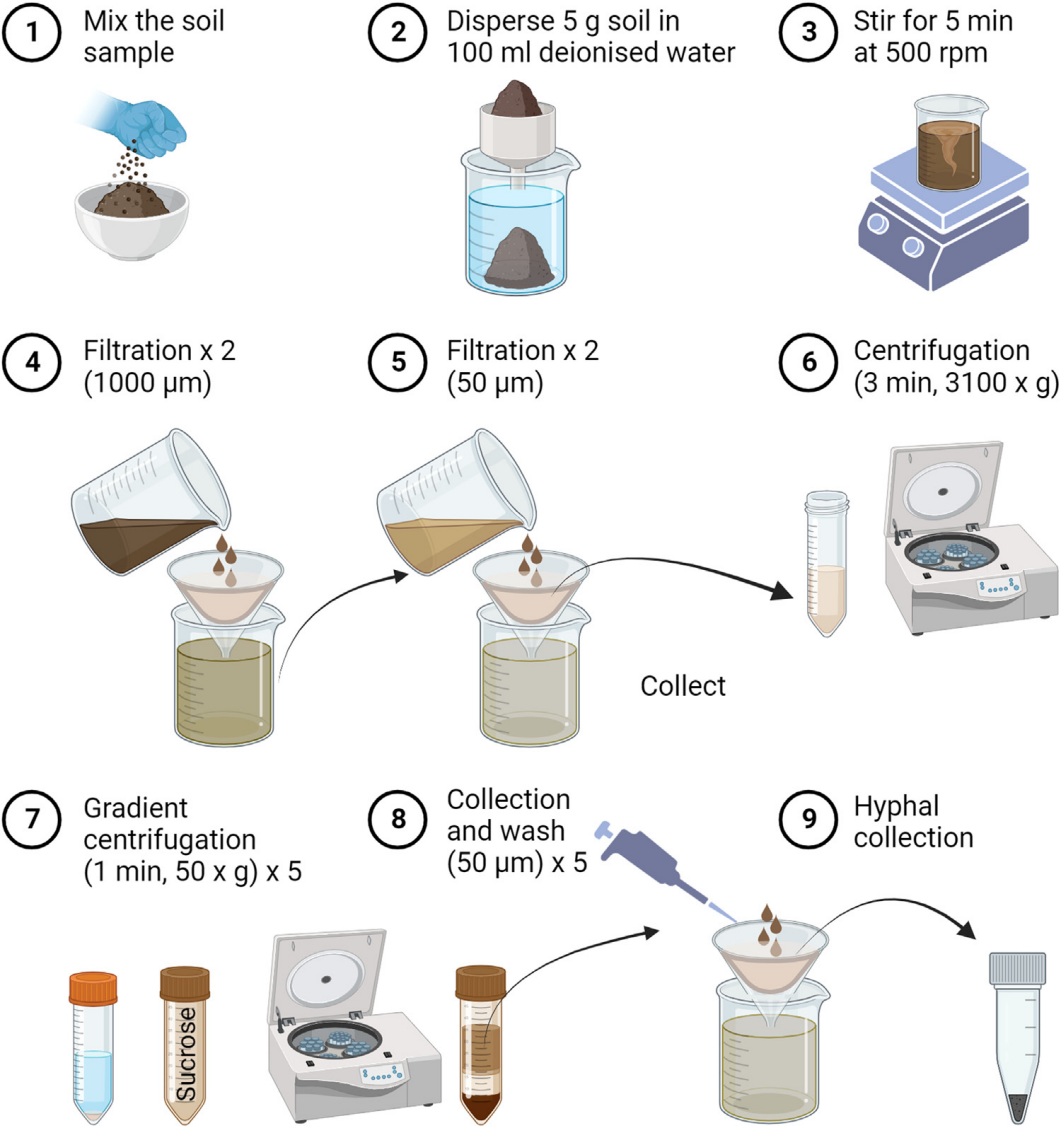
Schematic diagram of the procedure for soil mycelium extraction.

A soil sample (5.0 g) was dispersed in 100 ml deionised water using a bar stir on a magnetic stirrer (IKA Kombimag® RCT, IKA Labortechnik, Staufen, Germany) at a speed of 500 rpm for 5.0 min (step 2 and 3, Fig. 1). The obtained solution was filtered in two consecutive steps. The first step involves 2x filtration on a nylon mesh cloth (Franz Eckert GmbH, Waldkirch, Germany) of 1000 µm pore size, with a thorough washing of the oversize particles with 100 ml deionised water (step 4, Fig. 1). In the second step, the filtrate consisting in the soil solution and the washing water was further 2x filtrated, using a metal sieve of 50.0 µm pore size, padded with a 50.0 µm pore size layer of nylon mesh (Franz Eckert GmbH, Waldkirch, Germany). In this step, the filtrate was discarded. The oversize particles, mainly consisting of the bulk mycelial stock, were collected into a 50 ml-Falcon tube by a careful rising with 25.0 ml deionised water using a pipette (step 5, Fig. 1). The Falcon tubes containing mycelium were kept at 4 °C for several hours until other samples were processed, then centrifuged for 3.0 min at 3100 x g (Eppendorf centrifuge 5810R, Eppendorf GmbH, Hamburg, Germany). The centrifugation speed and time were adjusted to find no hyphae in the supernatant by the microscopic observation. The supernatant was discarded (step 6, Fig 0.1). The pellet was dispersed by vigorously hand shaking into 50.0 ml of 45.5 % sucrose solution and subsequently centrifuged at 50 x g for 1.0 min (step 7, Fig. 1). The supernatant was immediately collected and pipetted onto a three-layered 50.0 µm pore size nylon mesh (Franz Eckert GmbH, Waldkirch, Germany; step 8, Fig.1). The step of pellet dispersal into sucrose solution, followed by centrifugation, and the supernatant collection was repeated five times. The remaining pellet of soil residues was discarded. The particles collected on the nylon mesh were thoroughly washed with deionised water until the sucrose solution was rinsed out. Afterwards, they were carefully scraped off the mesh and collected into a 2.0 ml Eppendorf tube (step 9, Fig.1).

### Measurement of hyphal length

The hyphal length was measured in the initial soil, the extracted mycelium, and all discarded fractions of the mycelium extraction procedure.

To prepare the fungal hyphae for microscopic observation, a modified protocol based on [35,36] was followed. The soil sample (5.0 g) was suspended overnight in 200 ml of water mixed with 31.0 ml 35 % sodium hexametaphosphate solution. The soil suspension was then thoroughly stirred with a glass rod, filled with water to a final volume of 250 ml, and sonicated in a water-bath sonicator (Sonorex Super RK 510 H, Bandelin electronic GmbH, Berlin, Germany) at 320 W for two minutes intended to break up the soil aggregates. To cut the hyphae into shorter pieces, the soil suspension was blended for 9.0 s at 8000 rpm speed using Ultra Turrax T25 (IKA Labortechnik, Staufen, Germany). The beaker containing the soil suspension was placed on a magnetic stir plate (IKA Kombimag® RCT, IKA Labortechnik, Staufen, Germany) and stirred at high and then to a lower speed to allow soil particles to settle to the bottom of the beaker. During that time, an aliquot of 1.0 µl of the suspension was taken with a pipette from halfway between the beaker edge and bottom of the vortex and transferred into the central 16 x square of an Improved Neubauer chamber of 0.1 mm depth (VWR, Darmstadt, Germany). To measure the hyphal length of the collected mycelium and the discarded soil pellet, samples of mycelium and soil residues were suspended in 250 ml water and sonicated, blended and stirred as described above. The procedure negated the need for the overnight soil-soaking step. The Neubauer Improved chamber was observed under a microscope (Zeiss Primo Star, Zeiss, Jena, Germany) at 10 x magnification.

Using the gridline intersect method, hyphal intersections of all 48 vertical and 48 horizontal lines of the central 16 x square Neubauer Improved chamber were counted.

The hyphal length (H) was calculated by following Newman's formula [37].

$$H=\frac{\pi *N*A}{2L}$$

Where:

*N* = number of intersections, *A* = area of gridding, *L* = total line length of the gridded square

### DNA isolation and amplification

Genomic DNA was isolated from freeze-dried samples of soil-extracted mycelium using the Innu PREP Plant DNA kit (Analytik Jena, Jena, Germany) according to the manufacturer's protocol. Internal transcribed spacer (ITS) fungal rDNA region was amplified using the fungal-specific pair of the forward ITS1F (CTTGGTCATTTAGAGGAAGTAA) and reverse ITS4 (TCCTCCGCTTATTGATATGC) primers [38]. To check for positive amplification, 3.0 µl of the PCR products were mixed with 0.5 µL DNA loading dye GelRed (Biotium, Inc., Fremont, CA), loaded on 1.5 % agarose gel in TAE(Tris-acetate-EDTA) buffer, at 120 Vs for 35 min, and visualised under UV light (Fluor-STM multi-imager; Bio-Rad, Munich, Germany). A DNA ladder solution (Biotium, Inc., Fremont, CA) and positive and negative controls were also loaded in the gel to estimate the size of PCR products and for reference.

### Calculations and statistical analyses

The efficiency of the mycelium extraction procedure was defined as follows:

$$\text{Relative}\;\text{extraction}\;\text{efficiency}=\frac{\text{Total}\;\text{hyphal}\;\text{length}\;\text{in}\;\text{the}\;\text{collected}\;\text{mycelium}}{\text{Total}\;\text{hyphal}\;\text{length}\;\text{in}\;\text{the}\;\text{initial}\;\text{soil}\;\text{sample}}*100$$

Statistical analyses were performed using R version 4.2.1 (The R Foundation for Statistical Computing Platform 2022). Statistically significant differences between hyphal lengths of different soil fractions in the three regions were tested by Kruskal-Wallis one-way analysis of variance by ranks, followed by the post hoc Nemenyi's test using the function posthoc.kruskal.nemenyi.test in PMCMR package [39]. Fig. 1 was created with BioRender.com.

## Method validation

**Fig. 2:**
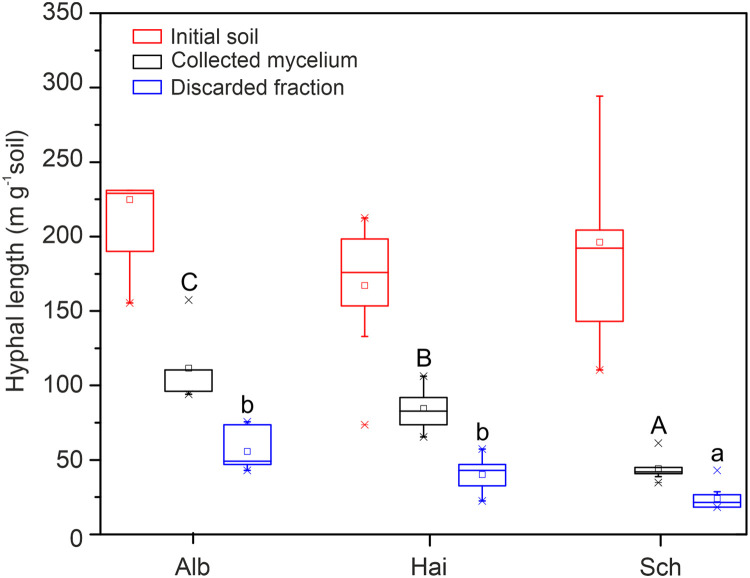
Hyphal length in the initial soil from where the mycelium was extracted, extracted mycelium, and discarded soil pellet at the end of the procedure. Soil samples were collected in three regions, Swabian Alb (Alb), Hainich-Dün (Hai), and Schorfheide-Chorin (Sch). Uppercase letters represent the statistical differences between the hyphal lengths of extracted mycelium; Lowercase letters represent the statistical differences between the hyphal lengths of discarded soil residues (*n* = 7 for Alb, *n* = 10 for Hai, and Sch).

Relative extraction efficiency (%) of soil mycelium, when the initial soil represented 100 % recovery, was estimated to 49.6 ± 3.5 % in Alb, 50.6 ± 2.3 % in Hai, and 22.5 ± 1.1 % in Sch soil samples. These results are in the range of those obtained by Bingle and Paul [30], who used glycerol density gradient centrifugation to obtain soil particle-free hyphae. Although the total hyphal length measured in the initial soil did not vary between soils of the three regions (Fig. 2), the hyphal length of extracted mycelium was significantly higher in the clay soils of Alb and Hai than in loamy sand soils of Sch (Fig. 2). In Alb soil, extracted mycelium, mean hyphal length value was about 110 m *g*^−1^, while in Hai soil was 80 m *g*^−1^ following the trend of a higher amount of clay found in Alb soils (496 g kg^−1^) compared with Hai soils (301 g kg^−1^, Table 1, [40]).

**Fig. 3:**
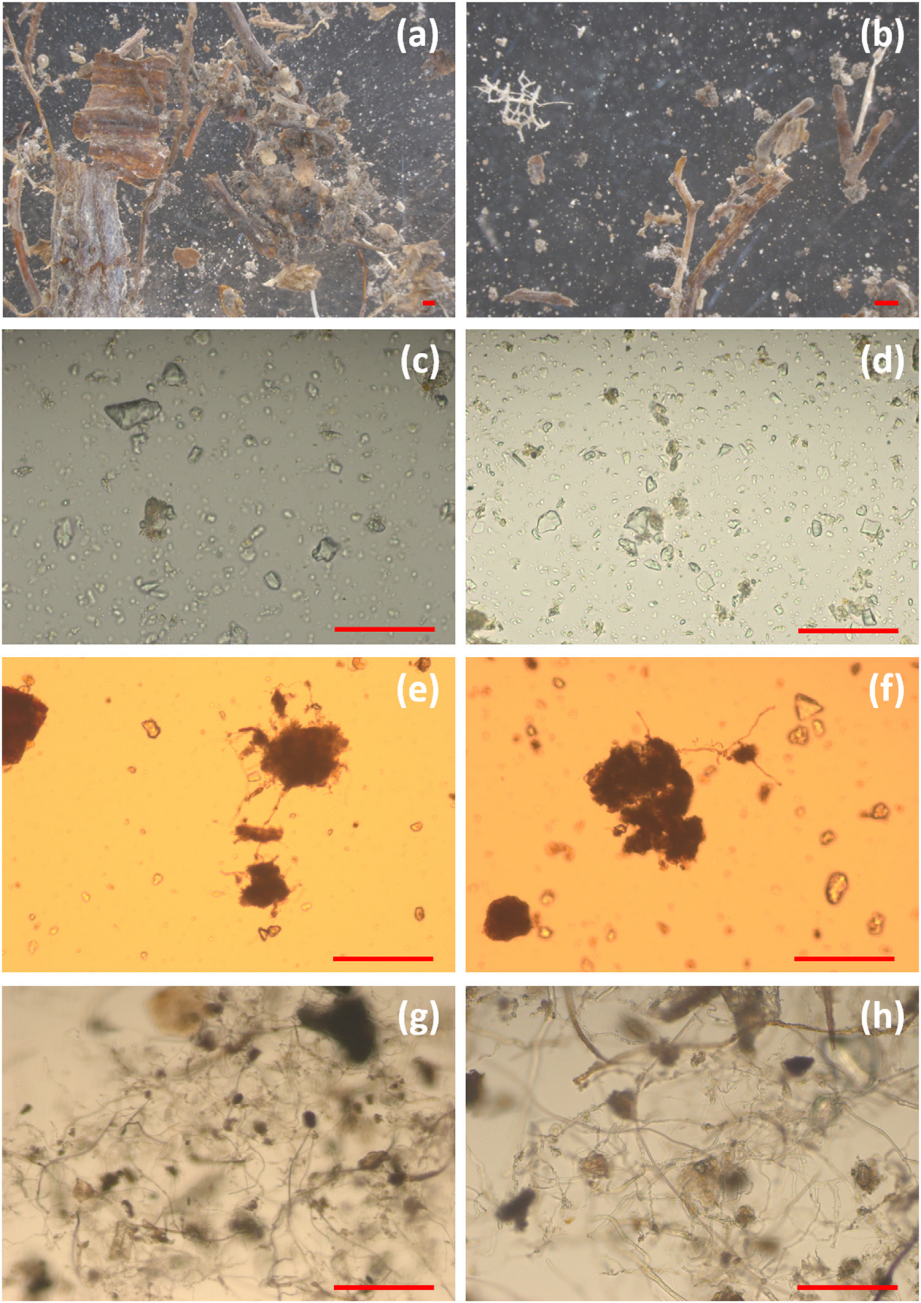
Microscopic pictures of oversized particles (>1000 µm) filtered in step 4 (a, b), discarded filtrate (<50.0 µm) filtered in step 5 (c, d), discarded soil pellet from sucrose gradient centrifugation in step 8 (e, f), and extracted mycelium in step 9 (g, h). The left-hand column shows Swabian Alb (Alb) samples, and the right-hand column samples from Schorfheide-Chorin (Sch). The scale bar represents 100 µm.

To recover the values found in the initial soil, hyphal lengths were measured in all discarded and collected fractions in the mycelium extraction procedure. However, hyphal measurement was not achievable in the oversize particle fraction in step 4 (1000 µm filtration, Fig. 1), which contained numerous root fragments rich in fungal structures of mycorrhizal root tips or pieces of the ectomycorrhizal mantle, and soil or sand particles carrying an unspecific amount of hyphae (Fig. 3a and b). The filtrates in steps 5 and 9 (Fig. 1) contained no hyphae (Fig. 3c and d). In the very last discarded soil pellet resulted from sucrose gradient centrifugation (step 8, Fig. 1), the hyphal length recovery was about 25 % (5 m *g*^−1^) in Alb and Hai soils, and 12 % (2 m *g*^−1^) in Sch soils (Fig. 2).

We assume that the remaining percentages of not recovered hyphae of 25% in Alb and Hai soils, and 65 % in Sch, were represented by hyphae lost in the oversize particle fraction from step 4 (Fig. 1). In this fraction, the hyphae can be either attached to the root fragments or other plant debris or attached to the soil particles. The latter situation is less probable because the clay aggregates disperse during stirring (step 1, Fig. 1), liberating the hyphae. During water immersion, the sand grains drop out of the hyphal biomass aggregates by 90 %, with almost 100 % release under stirring [41]. Therefore, a pre-step of removing the root fragments and other debris using tweezers under a stereomicroscope may increase the method efficiency to 67 ± 2 % in Alb, 68 ± 2 % in Hai, and 65 ± 1 % in Sch. Many studies use this removal step in preparing the soil samples for DNA isolation [42], [43], [44], [45]. The presence of mycorrhizal root tips in the soil samples dramatically biased the community composition of soil fungi. It has often been reported that sequencing the DNA isolated from root tips and soil collected at exactly the same spot results in two distinct fungal communities [27,46,47].

In the literature, it is commonly reported that the hyphal diameter ranges between 1 and 30 µm [48]. We found hyphae of 1–20 µm diameter (data not shown). However, in the soil, mycelium hyphae appear closely connected, and their length was greater than > 50 µm [49]. Therefore, the soft gravity filtration through multiple layers of 50 µm pore size mesh, instead of using a mesh of smaller pore size that requires a vacuum filtration, resulted in the maximal hyphal yield. Because all hyphae remained intertwined with one another in the mesh filter, we found none in the discarded solutions (steps 5 and 9, Figs. 1 and 3c, d).

We found no differences in the method efficiency between the silty clay (Alb) and silty clay loam soil (Hai), which is in accordance with Bingle and Paul [30]. However, in the absence of the pre-step of root fragments removal, the extraction efficiency was about two times higher in the clay relative to loamy sand soil (Sch). We explain this situation through the higher number of hyphae lost through when filtered with the oversize > 1000 µm root debris, which were more abundant in the sand than in clay soils. This is because, in temperate forests, beech trees present 35 % higher fine-root production [50], three times higher fine-root mass, and a ten-fold higher fine-root growth rate on sand than on clay soils [51]. If the pre-step of removing root fragments under a stereo microscope is carried out, this method will achieve a similar efficiency in different soil types.

**Fig. 4:**
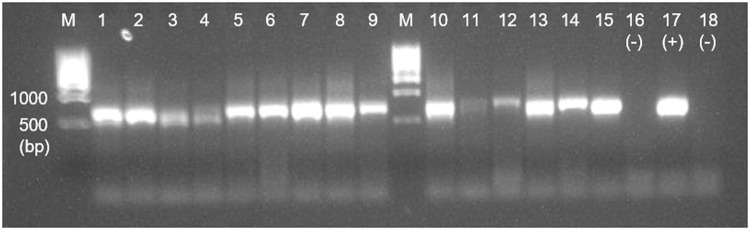
DNA electrophoresis of ITS1-F and ITS4 PCR products from soil-extracted mycelium. Lanes M, kilobase DNA ladder; lanes 1–5, Hainich-Dün (Hai) samples; lanes 6–10, Swabian Alb (Alb) samples; lanes 11–15, Schorfheide-Chorin (Sch) samples; lanes 16 and 18 (-), negative control; and lane 17 (+), positive control.

The extraction procedure used no chemical substances, preventing potential DNA isolation and amplification impairments. The extracted DNA from the collected soil mycelium was used to amplify the fungal ITS region. Electrophoretic separation of PCR products showed a high quality of the DNA yield in all soil types (Fig. 4). Moreover, the number of DNA reads obtained from these samples employing Illumina sequencing was >12,000 per sample (data not shown).

The soil mycelium extraction yielded a relatively soil-free bulk mycelium in an efficient (> 65 %), simple, fast, and low-cost procedure. Compared with the previously available method of Bingle and Paul [30], this improved protocol reduced the time needed for filtrations by five times and centrifugation from 40 min to 1 min. It enables handling a large number of samples and uses no chemical compounds, which may hamper further analyses. Thus, the extracted bulk soil mycelium can be used for DNA isolation, refining the DNA sequencing outcome to the specific soil-localized fungal communities, for analysis of mycelium chemical composition, and exploration of hyphosphere microbial niche. The method provides the means of investigation the functional aspects of soil fungi that may answer questions about their role in nutrient cycling, soil C sequestration, and climate change mitigation in terrestrial ecosystems.

## CRediT authorship contribution statement

**Abdallah Awad:** Methodology, Investigation, Visualization, Validation, Writing – original draft. **Rodica Pena:** Conceptualization, Resources, Supervision, Writing – original draft, Funding acquisition.

## Declaration of Competing Interest

The authors declare that they have no known competing financial interests or personal relationships that could have appeared to influence the work reported in this paper.

## Data Availability

Data will be made available on request.

## References

[1] Treseder K.K., Lennon J.T. (2015). Fungal traits that drive ecosystem dynamics on land. Microbiol. Mol. Biol. Rev..

[2] Baldrian P. (2017). Forest microbiome: diversity, complexity and dynamics. FEMS Microbiol. Rev..

[3] Simpson A.J., Song G., Smith E., Lam B., Novotny E.H., Hayes M.H.B. (2007). Unraveling the structural components of soil humin by use of solution-state nuclear magnetic resonance spectroscopy. Environ. Sci. Technol..

[4] Clemmensen K.E., Bahr A., Ovaskainen O., Dahlberg A., Ekblad A., Wallander H. (2013). Roots and associated fungi drive long-term carbon sequestration in boreal forest. Science.

[5] Chen J., Heikkinen J., Hobbie E.A., Rinne-Garmston K.T., Penttilä R., Mäkipää R. (2019). Strategies of carbon and nitrogen acquisition by saprotrophic and ectomycorrhizal fungi in Finnish boreal Picea abies-dominated forests. Fungal Biol..

[6] Pena R. (2016). Molecular Mycorrhizal Symbiosis.

[7] Joergensen R.G., Wichern F. (2008). Quantitative assessment of the fungal contribution to microbial tissue in soil. Soil Biol. Biochem..

[8] Hagenbo A., Clemmensen K.E., Finlay R.D., Kyaschenko J., Lindahl B.D., Fransson P. (2017). Changes in turnover rather than production regulate biomass of ectomycorrhizal fungal mycelium across a Pinus sylvestris chronosequence. New Phytol..

[9] Hagenbo A., Piñuela Y., Castaño C., Martínez de Aragón J., de-Miguel S., Alday J.G. (2021). Production and turnover of mycorrhizal soil mycelium relate to variation in drought conditions in Mediterranean Pinus pinaster, Pinus sylvestris and Quercus ilex forests. New Phytol..

[10] Awad A., Majcherczyk A., Schall P., Schröter K., Schöning I., Schrumpf M. (2019). Ectomycorrhizal and saprotrophic soil fungal biomass are driven by different factors and vary among broadleaf and coniferous temperate forests. Soil Biol. Biochem..

[11] Wang F., Zhang L., Zhou J., Rengel Z., George T.S., Feng G. (2022). Exploring the secrets of hyphosphere of arbuscular mycorrhizal fungi: processes and ecological functions. Plant Soil.

[12] Frey-Klett P., Chavatte M., Clausse M.L., Courrier S., Le Roux C., Raaijmakers J. (2005). Ectomycorrhizal symbiosis affects functional diversity of rhizosphere fluorescent pseudomonads. New Phytol..

[13] Uroz S., Calvaruso C., Turpault M.P., Pierrat J.C., Mustin C., Frey-Klett P. (2007). Effect of the mycorrhizosphere on the genotypic and metabolic diversity of the bacterial communities involved in mineral weathering in a forest soil. Appl. Environ. Microb..

[14] Emmett B.D., Lévesque-Tremblay V., Harrison M.J. (2021). Conserved and reproducible bacterial communities associate with extraradical hyphae of arbuscular mycorrhizal fungi. ISME J..

[15] Rozmoš M., Bukovská P., Hršelová H., Kotianová M., Dudáš M., Gančarčíková K. (2022). Organic nitrogen utilisation by an arbuscular mycorrhizal fungus is mediated by specific soil bacteria and a protist. ISME J..

[16] Tedersoo L., Bahram M., Zinger L., Nilsson R.H., Kennedy P.G., Yang T. (2022). Best practices in metabarcoding of fungi: from experimental design to results. Mol. Ecol..

[17] Lindahl B.D., Kuske C.R. (2013). The Ecological Genomics of Fungi.

[18] Hawksworth D.L., Lücking R. (2017). Fungal diversity revisited: 2.2 to 3.8 million species. Microbiol. Spectr..

[19] Leibold M.A., Holyoak M., Mouquet N., Amarasekare P., Chase J.M., Hoopes M.F. (2004). The metacommunity concept: a framework for multi-scale community ecology. Ecol. Lett..

[20] Dickie I.A., Boyer S., Buckley H.L., Duncan R.P., Gardner P.P., Hogg I.D. (2018). Towards robust and repeatable sampling methods in eDNA-based studies. Mol. Ecol. Resour..

[21] Nilsson R.H., Anslan S., Bahram M., Wurzbacher C., Baldrian P., Tedersoo L. (2019). Mycobiome diversity: high-throughput sequencing and identification of fungi. Nat. Rev. Micro..

[22] Zinger L., Bonin A., Alsos I.G., Bálint M., Bik H., Boyer F. (2019). DNA metabarcoding—Need for robust experimental designs to draw sound ecological conclusions. Mol. Ecol..

[23] Tedersoo L., Bahram M., Põlme S., Kõljalg U., Yorou N.S., Wijesundera R. (2014). Global diversity and geography of soil fungi. Science.

[24] Tedersoo L., Mikryukov V., Anslan S., Bahram M., Khalid A.N., Corrales A. (2021). The global soil mycobiome consortium dataset for boosting fungal diversity research. Fungal Divers..

[25] Bahram M., Hildebrand F., Forslund S.K., Anderson J.L., Soudzilovskaia N.A., Bodegom P.M. (2018). Structure and function of the global topsoil microbiome. Nature.

[26] Landeweert R., Leeflang P., Smit E., Kuyper T. (2005). Diversity of an ectomycorrhizal fungal community studied by a root tip and total soil DNA approach. Mycorrhiza.

[27] Genney D.R., Anderson I.C., Alexander I.J (2006). Fine-scale distribution of pine ectomycorrhizas and their extramatrical mycelium. New Phytol..

[28] Wallander H., Ekblad A., Godbold D.L., Johnson D., Bahr A., Baldrian P. (2013). Evaluation of methods to estimate production, biomass and turnover of ectomycorrhizal mycelium in forests soils – A review. Soil Biol. Biochem..

[29] Carini P., Marsden P.J., Leff J.W., Morgan E.E., Strickland M.S., Fierer N. (2016). Relic DNA is abundant in soil and obscures estimates of soil microbial diversity. Nat. Microbiol..

[30] Bingle W.H., Paul E.A. (1986). A method for separating fungal hyphae from soil. Can. J. Microbiol..

[31] Frankland J.C., Dighton J., Boddy L. (1990). Methods in Microbiology.

[32] Johnson S.R., Martin D.H., Cammarata C., Morse S.A. (1995). Alterations in sample preparation increase sensitivity of PCR assay for diagnosis of chancroid. J. Clin. Microbiol..

[33] Dames S., Bromley L.K., Herrmann M., Elgort M., Erali M., Smith R. (2006). A single-tube nucleic acid extraction, amplification, and detection method using aluminum oxide. J. Mol. Diagn. JMD.

[34] Fischer M., Bossdorf O., Gockel S., Hänsel F., Hemp A., Hessenmöller D. (2010). Implementing large-scale and long-term functional biodiversity research: the biodiversity exploratories. Basic Appl. Ecol..

[35] Addy H., McGonigle T., Brundrett M, Melville L., Peterson L. (1994). *Practical Methods in Mycorrhiza Research*.

[36] Miller R., Reinhardt D.R., Jastrow J. (1995). External hyphal production of vesicular-arbuscular mycorrhizal fungi in pasture and tallgrass prairie communities. Oecologia.

[37] Newman E.I. (1966). A method of estimating the total length of root in a sample. J. Appl. Ecol..

[38] Horton T.R., Bruns T.D. (2001). The molecular revolution in ectomycorrhizal ecology: peeking into the black-box. Mol. Ecol..

[39] Pohlert T. (2014). The pairwise multiple comparison of mean ranks package (PMCMR). R Package.

[40] Solly E.F., Schöning I., Boch S., Kandeler E., Marhan S., Michalzik B. (2014). Factors controlling decomposition rates of fine root litter in temperate forests and grasslands. Plant Soil.

[41] Went F.W., Stark N. (1968). The biological and mechanical role of soil fungi. Proc. Natl. Acad. Sci. U. S. A..

[42] Chen D.M., Cairney J.W.G. (2002). Investigation of the influence of prescribed burning on ITS profiles of ectomycorrhizal and other soil fungi at three Australian Sclerophyll forest sites. Mycol. Res..

[43] Dickie I.A., Xu B., Koide R.T. (2002). Vertical niche differentiation of ectomycorrhizal hyphae in soil as shown by T-RFLP analysis. New Phytol..

[44] Koide R.T., Xu B., Sharda J. (2005). Contrasting below-ground views of an ectomycorrhizal fungal community. New Phytol..

[45] Simmons T., Caddell D.F., Deng S., Coleman-Derr D. (2018). Exploring the Root Microbiome: extracting Bacterial Community Data from the Soil, Rhizosphere, and Root Endosphere. J. Vis. Exp. JoVE.

[46] Kjøller R. (2006). Disproportionate abundance between ectomycorrhizal root tips and their associated mycelia. FEMS Microbiol. Ecol..

[47] Peintner U., Iotti M., Klotz P., Bonuso E., Zambonelli A. (2007). Soil fungal communities in a Castanea sativa (chestnut) forest producing large quantities of Boletus edulis sensu lato (porcini): where is the mycelium of porcini?. Environ. Microbiol..

[48] Islam M.R., Tudryn G., Bucinell R., Schadler L., Picu R.C. (2017). Morphology and mechanics of fungal mycelium. Sci. Rep..

[49] Leake J., Johnson D., Donnelly D., Muckle G., Boddy L., Read D. (2004). Networks of power and influence: the role of mycorrhizal mycelium in controlling plant communities and agroecosystem functioning. Can. J. Bot..

[50] Hertel D., Strecker T., Müller-Haubold H., Leuschner C. (2013). Fine root biomass and dynamics in beech forests across a precipitation gradient – is optimal resource partitioning theory applicable to water-limited mature trees?. J. Ecol..

[51] Weemstra M., Sterck F.J., Visser E.J.W., Kuyper T.W., Goudzwaard L., Mommer L. (2017). Fine-root trait plasticity of beech (Fagus sylvatica) and spruce (Picea abies) forests on two contrasting soils. Plant Soil.

